# Blind people can actively manipulate virtual objects with a novel tactile device

**DOI:** 10.1038/s41598-023-49507-1

**Published:** 2023-12-21

**Authors:** Mariacarla Memeo, Giulio Sandini, Elena Cocchi, Luca Brayda

**Affiliations:** 1 Robotics, Brain and Cognitive Sciences Department Now With Center for Human Technologies, Fondazione Istituto Italiano di Tecnologia, Via Enrico Melen 83, Genoa, Italy; 2 Robotics, Brain and Cognitive Sciences Department, Fondazione Istituto Italiano di Tecnologia, Via Enrico Melen 83, Genoa, Italy; 3 Istituto David Chiossone per Ciechi e Ipovedenti Onlus, Geona, Italy; 4 Acoesis srl, Via Enrico Melen 83, Genoa, Italy; 5 Nextage srl, Piazza della Vittoria 12, Genova, Italia

**Keywords:** Biomedical engineering, Quality of life, Touch receptors, Information technology, Electrical and electronic engineering

## Abstract

Frequently in rehabilitation, visually impaired persons are passive agents of exercises with fixed environmental constraints. In fact, a printed tactile map, i.e. a particular picture with a specific spatial arrangement, can usually not be edited. Interaction with map content, instead, facilitates the learning of spatial skills because it exploits mental imagery, manipulation and strategic planning simultaneously. However, it has rarely been applied to maps, mainly because of technological limitations. This study aims to understand if visually impaired people can autonomously build objects that are completely virtual. Specifically, we investigated if a group of twelve blind persons, with a wide age range, could exploit mental imagery to interact with virtual content and actively manipulate it by means of a haptic device. The device is mouse-shaped and designed to jointly perceive, with one finger only, local tactile height and inclination cues of arbitrary scalar fields. Spatial information can be mentally constructed by integrating local tactile cues, given by the device, with global proprioceptive cues, given by hand and arm motion. The experiment consisted of a bi-manual task, in which one hand explored some basic virtual objects and the other hand acted on a keyboard to change the position of one object in real-time. The goal was to merge basic objects into more complex objects, like a puzzle. The experiment spanned different resolutions of the tactile information. We measured task accuracy, efficiency, usability and execution time. The average accuracy in solving the puzzle was 90.5%. Importantly, accuracy was linearly predicted by efficiency, measured as the number of moves needed to solve the task. Subjective parameters linked to usability and spatial resolutions did not predict accuracy; gender modulated the execution time, with men being faster than women. Overall, we show that building purely virtual tactile objects is possible in absence of vision and that the process is measurable and achievable in partial autonomy. Introducing virtual tactile graphics in rehabilitation protocols could facilitate the stimulation of mental imagery, a basic element for the ability to orient in space. The behavioural variable introduced in the current study can be calculated after each trial and therefore could be used to automatically measure and tailor protocols to specific user needs. In perspective, our experimental setup can inspire remote rehabilitation scenarios for visually impaired people.

## Introduction

Cognitive mapping is the mental process enabling a person to acquire, code, store, recall, decode and manipulate data about a spatial layout^1^. The output of such a process is crystallized in a time-dependent mental representation, defined as an internal model based on functional relationships among meaningful and self-explanatory elements distributed in space^2^.

To localize these elements, they should be defined in terms of distance and direction features^1^. They can be coded either by reference to the agent’s own body or movements, i.e. using an egocentric frame of reference, or to some external framework, i.e. using an allocentric frame of reference^2^. Some studies have specifically investigated navigation abilities in congenitally blind individuals, revealing that lack of vision induces the generation of an egocentric, rather than allocentric representation of the environment. For instance, Noordzij and colleagues^3^ tested the ability to form a spatial mental model on the basis of allocentric and egocentric descriptions. Blind people performed better after listening to an egocentric than an allocentric description, while the opposite pattern was found in the sighted. Furthermore, Rieser and colleagues^4^ reported that blind individuals found it difficult to estimate Euclidean distances between locations, whereas they handled functional distance more easily. This finding might depend on the specific difficulty in generating a mental representation of multiple objects and their relationship; accordingly, blind participants seem to be less accurate than blindfolded sighted controls in pointing tasks^4^.

Spatial ability is evaluated through tests, which assess mental imagery skills and accuracy in spatial visualization. In general, such tests are related to the recall of environmental information^5,6^.

### Mental imagery

More in depth, mental imagery is a multi-component ability involving different processes: generation, which creates the image in the visual buffer; inspection, which allows identifying parts and relations within the image; and transformation, which allows image manipulation, for example, by rotating or translating it^7,8^. Generating and maintaining the representation of an object, and simultaneously figuring out what the object would look like if it was rotated^9^, is important in everyday life because it represents one of the basic elements underpinning our ability to re-orient in space^10^. This top-down process is in place every time a blind or visually impaired (BVI) person seeks to find his/her own location while touching a tactile map. This is, in principle, quite demanding, due to the substantial recruitment of the working memory. However, since it involves mental manipulation, it is found to be more advantageous for learning than a mere bottom-up exploration of the environment^11,12^. Some studies state that blind and visually impaired children show higher working memory capacity than sighted children^13^, suggesting that they can be involved in activities where the ability to simultaneously compare and store different information in short-term memory is required^14^. However, in studies that display spatial information to blind participants as auditory items^15^ or as maps made of elementary blocks^16^ or using programmable tactile displays^12^, the opposite pattern is found. Among other abilities, mental imagery training enhances Verbal Comprehension, Visual Perceptual Reasoning and Working Memory^17^.

### The role of manipulation

Although spatial skills are malleable, and training in spatial thinking is effective and durable^18^, the extent to which perception and manipulation (which includes perception) tasks can be used to elicit, or even train, mental imagery in visually impaired people, remains unclear.

Handling the elements of a map seems in fact to be beneficial per se, because learning *how to* develop a certain knowledge, rather than simply (*k*nowing that) the task should be completed in a certain way, is likely to facilitate the recall and use of such knowledge^11^. If the map is purely tactile, the spatial learning and the practical ability to orient in space are enhanced in BVI persons^19–21^: in orientation and mobility protocols, recognizing a map is usually done by matching the tactile reproduction with the existing environmental counterpart; map elements are recalled for taking decisions during navigation and to plan the route in advance^22,23^.

In absence of vision, interactivity facilitates the usability, of virtual tactile maps^24,25^. The interaction can be unilateral, i.e. when the information shared with the user is static^24^; or bilateral, i.e. when the user can actively modify the content. The second scenario involves the use of spatial skills as the ability to imagine and mentally transform spatial information^18^. Bilateral interactivity comes with the enhancement of spatial skills^26^. Targeted trainings can ameliorate the completion of spatial tasks, such as search and localization tasks, as described by Reynolds et al.^27^, where the authors find that our minds integrate with the environment in such a way that when the environment responds to our actions we are able to synchronize with it.

### Virtual learning setups

Several studies suggest that virtual setups have a number of advantages when administered to BVI people: they help with less biased and more versatile stimulation tasks; they are economical and flexible in the design of realistic experimental settings; they allow online recording of the participants’ behaviour^28,29^ and can be structured as serious games. For instance, Connors, Merabet and colleagues used an Audio-based Environment Simulator (AbES) to explore a virtual building set^30,31^. Early blind participants succeeded in generating an accurate spatial mental representation and were able to transfer it into equally accurate navigation performance. Moreover, augmented reality systems offer an efficient solution guiding users in real scenarios. One example is the NAVIG system^32^, which uses spatialized semantic audio rendering to inform users about the route and the surrounding elements of a pre-set list of destinations.

Attempts to create purely tactile virtual environments with a unilateral interaction were done by Ziat^33^ and then by Rastogi, Prescher, Gutiérrez-Fernández, Lahav et al.^34–36^. In the first two studies^33,34^ participants explored the virtual environments by means of two Braille-cells displays and tested two tactile zooming techniques. Both methods of interaction implied rebuilding the boundaries of the picture with touch, zooming in the static picture, but they presented the same virtual content. Only in the last three studies^34–36^, the experimenters recruited BVI individuals. After long sessions of training, the participants were able to distinguish subtle details of virtual environments. A recent study examined the multisensory interaction of visually impaired people with virtual environments^37^ and demonstrated that the participants were not only able to create a cognitive map, but also to perform orientation tasks. This work is the result of previous studies, in which the authors found that providing appropriate spatial information through compensatory sensory channels helps with mental mapping^38^ and with successful performance in real tasks^39^.

### Orientation and mobility aspects

Rather than the mere presence or absence of vision, spatial skills can heavily depend on how BVI people acquire and build environmental information^2,40^. Therefore, when designing assistive technology for BVI persons, it is important to consider intrinsic or extrinsic aspects in training orientation abilities^41,42^, for example by disentangling strategy and performance^40^.

This approach allows to dissect performance, while seeking for the underlying cause of an observed behaviour^43^. It is important to keep training strategies flexible and adaptable to specific personal challenges, needs and learning skills. Virtual environments can provide this adaptability and may integrate current Orientation and Mobility (O &M) protocols^44^. Furthermore, performance on spatial updating tasks improves when either the amount of O &M training or experience in exploring tactile maps increase^2,22,45^.

To the best of our knowledge, no study so far investigated if and how, beyond interaction, manipulation of virtual-tactile maps could also be achieved. Specifically, we wondered if blind people were able not only to understand virtual tactile objects of a map but also to modify their position so that they could form a new arrangement, i.e. a new virtual object. Our study also aims at finding some accuracy cues at the behavioural and subjective levels that can help with measuring the manipulation process, by clarifying if *the way* a map is created can be a proxy of how well it is understood. We wanted the process of map editing to be measurable, with specific reference to how accurate and fast it was. We chose to split the editing process among the two hands, by attributing the sub-process of perception to one hand and the sub-process of action to the other.

### Small-area tactile displays

In this study, we chose to limit the sub-process of perception to a small-area display. Small-area displays have well-known benefits. They provide simple, cheap and lightweight aids, with an extra cost in terms of mental effort. The Optacon^46^, initially created with the purpose of helping visually impaired people read texts, has also been used to understand images. It has a camera that detects texts or images and an elaboration unit that converts them into tactile feedback (pin-array technology) on a stationary fingertip. Nowadays, it is mostly used for basic haptic research on tactile perception^47^ or the accuracy performances for map accessibility^48^.

Information gathered by one finger only triggers the continuous active hand-arm motion in order to integrate the perception on the fingertip with the proprioception, with the goal-directed process of recognizing tactile pictures. The following devices exploit this process of picture reconstruction through touch. Laterotactile display^49^ is a substitution device generating lateral skin deformation to convey the illusion of exploring 2D shapes on a flat surface. It approximates height information (the raised line) with tangential information, using a haptic illusion. The VT-player^50^ (VirTouch Ltd. Available) is a mouse-shaped device providing tactual information about the shapes and edges of a map with the use of two Braille cells. Both systems lack the possibility of delivering three-dimensional spatial content^51^.

Phantom and Falcon devices measure the hand position and exert a force vector based on the objects present in the virtual environment. The only pitfall is that accessibility to a virtual environment is not direct but mediated by a stylus or sphere. It is a limit because sensing the force-feedback on a single point through a stylus (forcing the hand to be in a ’grasp’ position) is not a natural way of interacting and of perceiving the shape of objects. However, they are effective in rehabilitative contexts, to render 3D surfaces^52^ and to create 3D virtual environments which support mental maps creation^38^ and the subsequent good performance in real space tasks^39^.

### Bi-manual manipulation

Several studies have shown that haptic tasks, even in virtual environments, can be ecologically kept bi-manual, as it frequently happens with real editing and manipulation activities^53,54^. Specifically, if the two hands work on independent sub-tasks^33,55^ that are each sequential^56^ and specialized^37,57^, then bi-manual interaction can improve cognitive efficiency^58^ and reduce the gap between novices and experts compared to the use of only one hand^59^. This fact is in-line with Guiard’s Kinematic Chain model, which describes the sequence of action during a bi-manual task with a focus on the role of each hand^60^: the model defines the roles of the Dominant Hand (DH) as opposed to the Non-Dominant Hand (NDH), in which the NDH precedes and acts as a frame of reference for the DH, which performs finer actions. Interestingly, the model allows DH and NDH to operate at different paces, something that has been shown to enhance the interaction with virtual environments^61^ when different tasks are assigned to the hands^62^.

## Results

### General linear models

This section describes the analyses of the *Manipulation experiment* with the description of the general linear models performed on the variables of Accuracy, Efficiency and Execution Time.

#### Accuracy

The Accuracy is analysed as a dependent variable to evaluate the potential effect of a set of independent variables, i.e. the predictors. The predictors were chosen with the perspective of help in the rehabilitation context: the Efficiency Ratio, the Execution Time and the Resolution of virtual environment. While Efficiency Ratio and Execution Time are continuous variables, Resolution is categorical, with three levels representing the resolutions of the matrices in each session (Resolution 1: 2 rows $\times$ 2 rows, Resolution 2: 2 rows $\times$ 3 columns and Resolution 3: 3 rows $\times$ 3 columns). The Accuracy was modelled with a Poisson distribution, i.e. a discrete distribution expressing the probability of a given number of events occurring in a fixed interval of time. As depicted in Table 1, there was a significant effect of the Efficiency ratio on Accuracy while Resolution barely reached the significance, whereas Execution Time and Gender did not predict it.

**Table 1:** Accuracy. Significant (p < 0.05) p-values in bold, mildly significant values in italic.

Effect of	Df	Estimate	Std. error	z	Pr (>|z|)
Efficiency ratio	1	1.86	0.44	4.22	**0.00002**
Resolution	2	$-0.11$	0.05	$-1.96$	*0.05*
Execution time	1	$-0.001$	0.001	$-1.12$	0.26
Gender	1	0.02	0.05	0.37	0.71

**Figure 1:**
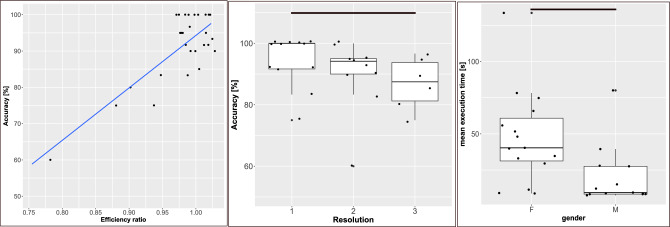
(Left) Linear prediction of the Accuracy in building virtual objects. Box-plots and models are shown with raw data superimposed. The black lines link two significantly different conditions. The Accuracy was predicted by the Efficiency Ratio. (Center) Percentage of the Accuracy in building virtual objects depending on the Resolution of the virtual environment. (Right) Execution Time needed to build virtual objects depending on the Gender of participants.

To further evaluate the relation between Accuracy and significant variables, the following analyses were performed. In the case of Efficiency Ratio, a simple linear regression model was used. The results showed that Accuracy and Efficiency Ratio were linked by a linear relation: R$^2$ = 0.66, F(1,26) = 51.22 and p = 0.0000001 (see Fig. 1 (Left)).

Wilcoxon post-hoc test revealed a significant difference in Accuracy values between the first and the third Resolution (W = 56.5, p = 0.02) as depicted in Fig. 1. The overall mean Accuracy, across the three resolutions, was 90.5% , with a mean value of $94.4 \pm 8.2$ for Resolution 1, $90.2 \pm 11.7$ for Resolution 2 and $86.9\pm 8.5$ for Resolution 3. Therefore, Accuracy decreased while increasing the Resolution, which reflected the increasing difficulty of the task.

#### Execution time

The Execution Time was analysed as a dependent variable using a general linear model, with Gender, Resolution and the percentage of Mouse Use as possible predictors. Execution Time was modelled with a Student’s t-distribution, i.e. a continuous probability distribution arising when the mean of a normally distributed population is estimated, sample size is small and standard deviation unknown. Table 2 shows that only the participants’ Gender was a significant predictor of time required to accomplish the task. The average Execution Time was 34.9s $\pm 30.4$. In particular, women were slower than men in manipulating objects, needing respectively 47.7s $\pm 32.3$ (median of 40.4s) and 20.2s $\pm 20.6$ (median of 9.5s) on average. See Fig. 1 (Right) for further details.

**Table 2:** Execution time. Significant (p < 0.05) p-values in bold.

Effect of	Df	Estimate	Std. error	z	Pr (>|z|)
Gender	1	$-28.89$	12.06	$-2.39$	**0.02**
Mouse use	1	0.33	0.43	0.76	0.45
Resolution	2	$-20.78$	14.3	$-1.45$	0.16

#### Efficiency ratio

The Efficiency Ratio was also analysed with a general linear model. The independent variables in this case were Resolution of virtual environment, percentage of Mouse Use, Gender and Execution Time. The Efficiency Ratio was modelled with a Student’s t-distribution. The Efficiency Ratio had a median of 1.00 and an average 0.98 $\pm 0.05$. As shown in Table 3, none of the independent variables had a significant effect on the efficiency in solving the task. As a consequence, we were not allowed to proceed with the post-hoc analyses.

**Table 3:** Efficiency ratio. No significant predictions appear.

Effect	Df	Estimate	Std. error	z	Pr (>|z|)
Resolution	2	0.01	0.03	0.25	0.8
Mouse use	1	$-0.001$	0.001	$-0.74$	0.47
Gender	1	$-0.03$	0.03	$-1.21$	0.24
Execution time	1	$-0.0002$	0.0004	$-0.54$	0.59

#### System usability scale (SUS)

We collected the answers to the System Usability Scale questionnaire. The scores given by each participant were summed depending on the question number, multiplied by 2.5, then averaged, as done in a previous study^63^. The SUS scores ranged from 0 to 100. The final rate was 45. A score of 65 is considered as average acceptability value^64^, thus the system had low usability. Since the SUS was applied to a system composed by two sub-systems (the TAMO3 and the keyboard) we investigated the acceptability of the two sub-systems separately, with two additional items:

- I felt very confident using the TAMO3.
- I felt very confident using the keyboard.

The range of answers was 1–5, where higher values meant stronger agreement with the asked question. TAMO3 was rated with a score of 3.5 and the arrow keys with a score of 3.7, therefore they perceived those devices as likely usable.

#### Analysis of bimanual synchronization

**Figure 2:**
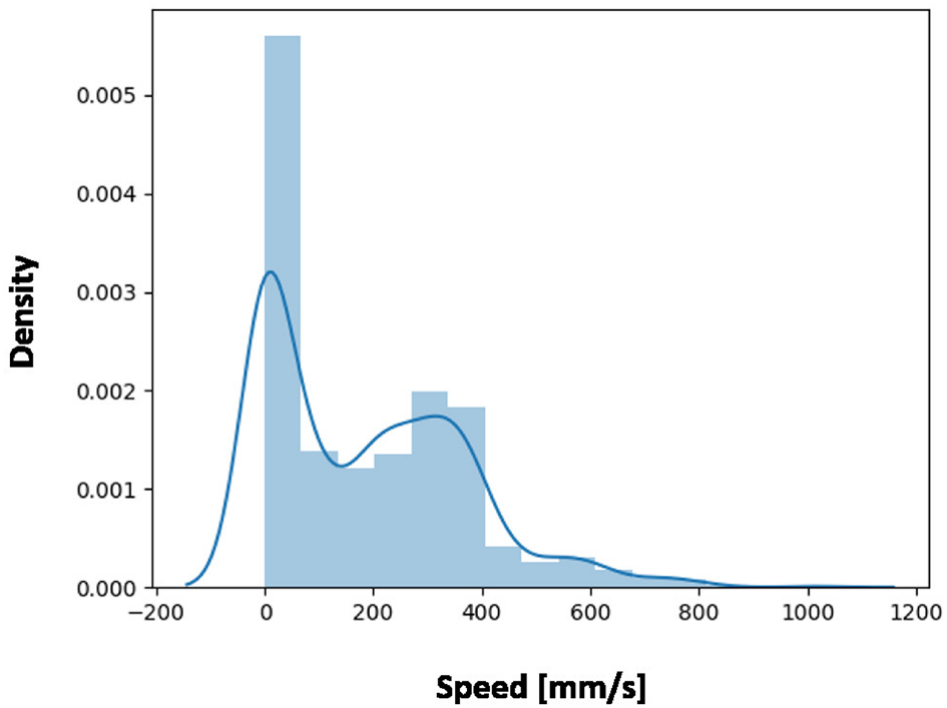
The histogram represents the frequency and density of distribution of the TAMO3 device movements during the key presses. The speed is calculated as the derivative of the TAMO3 position in the time samples when a keyboard key was clicked and the following sample, respectively.

We wondered if our bi-manual task, which in principle required two different sub-processes assigned to each hand, resulted in a purely asymmetric sequential scale of motion or not, i.e. if the dominant hand was acting only when the non-dominant hand was still and vice versa. Since one hand was moving the haptic device to perceive the position, shape and size of objects, we wondered if that hand was not moving while the participant was editing the map with the other hand. If that is true, the haptic device should be at zero velocity every time the button of the keyboard is pressed. The histogram in Fig. 2 depicts the frequencies and, the relative density distribution, of the TAMO3 speeds at the moment in which any key-press occurred. Although the dominant bin is at zero speed, very frequently the two hands are acting simultaneously, in every bin in which speed is greater than zero.

## Discussion

This study is motivated by the intent to understand if visually impaired people can exploit mental imagery to manipulate virtual content by means of a haptic device. To the best of our knowledge, it is the first study to make such an attempt.

The results show that the overall Accuracy in manipulating virtual objects was 90.5%, demonstrating that visually impaired people are able to handle the task not only at a basic but rather at a high performance level. This high performance was achieved with little or no training at all, with the cautious but reasonable assumption that the familiarization phase with the real setup implies little training.

### Efficiency is linearly correlated to Accuracy in solving the task

For practical reasons, it is important to trace and store the performances of the participants. Therefore, the Accuracy was chosen as a dependent variable for the first model. As predictors, the Efficiency Ratio was chosen as behavioural variable, while the Execution Time and the Resolution of the virtual environment were chosen as objective variables.

The Efficiency Ratio represents the amount of moves made by the participants with respect to the ideal minimum number of moves to complete each trial. The model showed that the Accuracy is positively correlated with the Efficiency Ratio: the more the participants are able to carefully choose (i.e. minimize) the number of moves to correctly build the final object, the more accurately they perform. The measure of efficiency could represent valuable information to quantitatively evaluate the trend of a rehabilitation protocol. By quantifying how efficiently participants interact with virtual objects, we can track their performance trends. Importantly, it could be used as a target measure for tailored exercises. By setting efficiency goals, rehabilitation exercises can be tailored to address individual needs and improve a participant’s ability to find the most efficient path to manipulate virtual objects. Virtual environments offer unique opportunities for individuals with visual impairments, as they can engage in spatial tasks, such as puzzle-solving, map exploration, and object manipulation, in a controlled and customizable setting. These experiences can be tailored to suit various levels of complexity and serve as a valuable tool for enhancing spatial skills and cognitive mapping.

Moreover, the Accuracy slightly decreases as the Resolution of the virtual environment increases, meaning that the resolution can be used in general to challenge the participants or match their abilities, by increasing the difficulty. Other means of increasing difficulty, not explored in this study, could also come from decreasing the available information about the virtual layout (no hint about initial object positions), avoiding the use of sound interactions and using spatial limits to signal invalid moves. Note that the Execution Time did not influence the Accuracy: this non-significant result is important because, at least in this task, manipulating objects faster does not guarantee reaching the task goal. Coupled with the positive effect of the Efficiency ratio, this result implies that strategy is more important than speed.

### Gender influences execution time

Since time in the rehabilitation context is important to identify sources of modulations in learning abilities, the possible predictors of Execution Time were a diverse and complementary set of variables. They consisted of a behavioural parameter (the percentage of time in which the TAMO3 was moved), one subjective parameter (the gender of the participants) and one objective parameter (the resolution of the virtual environment).

Only the Gender affected the Execution Time, so the necessary time to execute the task depends only on the intrinsic characteristics of the participants: to achieve similar performances, women and men need different execution times. The relation between Execution Time and Gender can be important to personalize exercise protocols.

Admittedly, the participants’ gender was not balanced in this study (five men, seven women), therefore the results should be interpreted with caution. However, in the literature, there are known gender differences that could support our outcomes. One possible source of difference is the perception of cognitive load. There is a gender-dependent perception that is more marked as the elements to be kept in working memory increases : the cognitive load perceived by women is generally higher than men’s perception^65,66^. Moreover, men are usually more flexible and successful at orientation tasks. In real and virtual environments, men can easily switch from an egocentric to an allocentric perspective, whereas women are more constrained by a given perspective^67^. Regarding standard spatial tests, men outperform women in mental rotation tasks, but men and women are similar in remembering the object locations^68^.

Then, the amount of time the mouse was used did not have an effect on how fast people completed the task. This was expected, since the TAMO3, in this experiment, was mainly used to verify the intended moves of the participants. The rendered objects were all of the same shape (parallelepipeds) and size (within the same resolution value, while the size varied across resolutions), with constant height profiles along both x and y axes. We can speculate that objects with varying height profiles could have had significant effects on the execution time.

For possibly the same reason, the resolution of virtual objects did not sufficiently modulate the execution time.

### Neither behavioural nor subjective variables influence efficiency

Additionally, we were interested in finding possible predictors of the efficiency ratio, which, in turn, we already found to be the sole predictor of Accuracy. The independent variables were a collection of predictors used in the previous models. We found that the efficiency was independent of subject-related variables (gender) or task-related variables (task difficulty, percentage of mouse use, execution time). Therefore, the ability to minimize the number of moves appears to be a pure measure that can not be inferred from any other kind of variable evaluated in this study. One possible explanation is that this skill originates from diverse levels of mental imagery and working memory across our participants, but we were not able to measure such variability in this work.

### System usability has room for improvement

The system, i.e. the TAMO3 and the keyboard, was evaluated with the System Usability questionnaire (SUS). The questionnaire gives a quantitative measure of subjective evaluations about how usable a system is. The setup used was rated with a low level of usability, possibly because it was the first exposure to it and only a minimum amount of training was provided. The relatively high score of the two additional items that separately evaluated the TAMO3 and the keyboard, may imply that the SUS on the whole system may have been negatively influenced not by the instruments, but by the task itself or by the difficulty, or novelty of the game.

The participants gave us suggestions about how to improve the task of the *Manipulation experiment* and they were mainly three:

- The dimensions of the mouse should be reduced in order to improve its usability and ergonomics. This adjustment would make it more comfortable for users to handle and operate the TAMO3;
- The mouse could be more autonomous while performing the experiment, providing the possibility to move the objects without using the keyboard;
- Additional reference sounds could be attached for each object, in order to distinguish them better. The auditory feedback can contribute to a more accessible and user-friendly experience by providing clearer cues for object identification. Semantic or spatialized sound offers a valuable possibility to create, in a future application, a virtual environment representing a virtual tactile map to be used in the O &M trainings.

These refinements can enhance the overall usability and effectiveness of the system, making it more user-centric and accessible. Note that all participants were frequent keyboard users and that they rated its usability with a value similar to that of TAMO3, when separately considered; therefore they perceived TAMO3 as acceptable as one of the most common PC input systems. This is encouraging from the perspective of using TAMO3 as a daily rehabilitation tool.

### Compliance with the Guiard’s bimanual Chain Model

We designed our setup by following the principles of Guiard’s Kinematic Chain model^60^ to ensure realism in the interaction with the environment. In this model, both hands act on asymmetric temporal-spatial scales of motion as if they were two motors connected in series. We checked if the bi-manual interaction in our experiment was congruent with the model. Figure 2 shows the speed of the dominant hand (DH) during the actions of the non-dominant hand (NDH). The multi-modal distribution consists of a component with positive speed and one when the hand does not move. The latter component demonstrates that the bi-manual task required sub-processes that are in part sequential, in line with Guiard’s model. In other words, our participants were—most of the time—moving the mouse while moving the virtual objects with the keyboard, but they also took the time to explore the new manipulated object after having pressed the keyboard button. Further analysis is necessary to investigate what motivated the participants to choose the first strategy (using both NDH and DH) or the second (stopping the NDH and using the DH).

However, the presence of a large component with positive speed needs a discussion focused on the haptic interaction of our task in the absence of vision. When bi-manually building objects, in virtual environments, the haptic feedback can be delivered to only one hand, without impairing the performance^69,70^. Additionally, since the accuracy is in general best when the NDH orients the target object^55^, we chose to deliver the perception of the virtual object to the DH and the position editing to the NDH. This is also consistent with the finding that the tasks assigned to the hands should be orthogonal in terms of cognitive effort, otherwise performance may decrease significantly, due to an effect of *division of attention*^71^. In cases were the attention is not too divided, two-handed interaction allows better integration of multiple sub-tasks at the cognitive level^72^. The temporal symmetry between the hands is also influenced by the task complexity. Several studies on bi-manual tracking tasks showed that, while increasing the difficulty, the need to divide the attention and the lack of visual integration led to a more sequential way of performing tasks^55,73–75^. This confirms that the perception and action sub-processes, in this study, are substantially orthogonal and one hand does not impair the other hand during the task.

### Contribution to rehabilitation protocols

This work demonstrates that visually impaired people can dynamically imagine and actively update mental maps, when objects are virtual. First, we showed that performance can be predicted both by a behavioural variable corresponding to the number of moves in the virtual matrix and, in part, by task difficulty. Second, men are faster than women, but time does not affect performance.

If we imagine casting this setup in a rehabilitation protocols, the practitioner could exploit the gender effect, by adjusting the time according to the gender, or by exploiting a measure of efficiency automatically given by the system after each trial: knowledge about efficiency can help to train and stimulate the acquisition of strategies to improve it, indirectly enhancing accuracy. In other words, rather than just focusing on improving performance, blind users may concentrate on a much more measurable and meaningful aspect, such as the strategy behind performance.

Although in our task we did not explicitly indicate the fixed virtual objects and the ’out-of-border’ sounds as landmarks and clues, they may have helped a lot in constructing the cognitive map of the action grid. To demonstrate if this is actually the case, one should explicitly measure how much the participants rely on virtual beacons, something that we have not done in this study.

Finally, this study was performed with no training, therefore we speculate that the participants may have rated the overall usability as low because they have never used a computer keyboard with a tactile mouse together, and that within a rehabilitation program, this difficulty could be easily overcome with training sessions.

## Methods

### Setup

In our experiment, the participants could perceive the shape and size of basic virtual objects, while at the same time being able to act on the objects by changing their position. The goal was to create a new and more complex object. The sub-process of perception was achieved with a haptic tool, which was also used to verify the new position of virtual objects after editing. The sub-process of action was achieved with four buttons on a keyboard, indicating the direction in which the basic objects had to be moved. As a consequence, the virtual scene was dynamic and could be edited by the participants.

**Figure 3:**
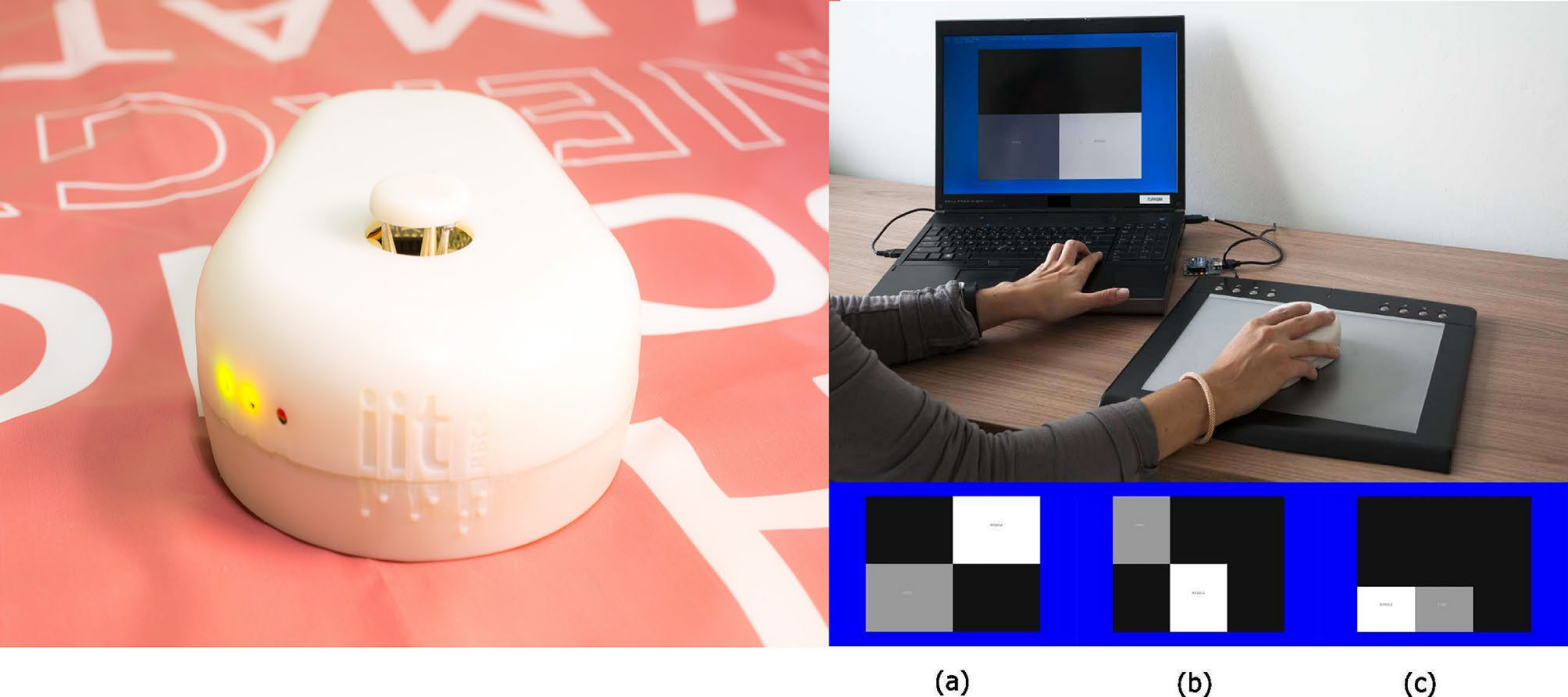
(Left) The TAMO3, the tactile device used for this study. (Right-Up) Experiment scenario: the interaction with both the arrow keys and TAMO3 with the left and right hands respectively. (Right-Down) The three resolutions of the virtual environment. Left to right: 2 rows $\times$ 2 columns, 2 rows $\times$ 3 columns and 3 rows $\times$ 3 columns. The environment and the objects were coloured to help the experimenter distinguish them. On the screen, the blue and the black portions are the background (with haptic height at 0mm), but objects can be moved only on black and not on blue portions; the objects are depicted in grey or white, (with haptic height at 15mm), but only white objects can be moved. The feedback from TAMO3 was the same for both objects. *Fixed* objects were coloured in gray, while *movable* ones were white and could be moved only into the black spaces.

The haptic tool was the TActile MOuse 3 (TAMO3), see Fig. 3 (Left), a mouse-shaped device able to deliver, to one finger only, the profile of a virtual surface. The participants were asked to explore and manipulate objects in a virtual environment, using the TAMO3 and a PC keyboard. The tactile interaction with the virtual objects was provided by the TAMO3 to the DH, while the objects were moved with the arrow keys of a keyboard by the NDH. The details about the operating principles of the TAMO3 haptic device can be found in our previous study^76^ (https://doi.org/10.6084/m9.figshare.21517359.v1).

### Participants

Twelve visually impaired volunteers (7 women) participated in this study. The sample was formed by 11 totally blind subjects and 1 partially sighted; among them, 9 were congenitally and 3 late blind (they lost their sight in adulthood). Their ages ranged from 11 to 61 years (28 ± 17 years old). All of them were naïve to the task and reported to be right-handed. The participants had no scars or other damages on the fingertip of their index finger of the DH. The protocol of the experiment was approved by the local Ethics Committee (Azienda Sanitaria Locale 3, Genoa) and procedures complied with the Declaration of Helsinki. Informed consent was obtained from all participants.

### Procedure

We investigated whether a sample of visually impaired participants could interact and manipulate a virtual environment. In this study, the environment is analogous to a map, i.e. an abstract representation of a generic portion of space arranged in a structured grid. Maps contain elements diversely classified and empty spaces in which objects can be potentially placed. The map encompasses only two objects, one is fixed and one that can be moved, both placed in a virtual grid at different resolutions. The manipulation involves haptic exploration of these objects from a top-view perspective.

The experiment, called *Manipulation experiment* from now on, was divided into three sessions with increasing difficulty. Figure 3 (upper part) shows the experimental setup. In each session, the virtual environment was composed of a *fixed* and a *movable* object, i.e. two parallelepipeds. The task was to bring the *movable* object next to the *fixed* in order to form a *target* object, i.e a bigger parallelepiped of the requested orientation. The orientation of the *target* parallelepiped could be either vertical (i.e. the centres of each object had the same X coordinate) or horizontal (i.e. the centres of each object had the same Y coordinate) according to the coordinates of the centres of both *fixed* and *movable* object.

Both *fixed* and *movable* objects were displayed in a matrix whose resolution changed depending on the session. From the first to the third session, the resolution of the virtual matrix was respectively 2 $\times$ 2 (2 rows and 2 columns), 2 $\times$ 3 and 3 $\times$ 3. Thus, the available positions of both objects increased from 4 to 9.

**Figure 4:**
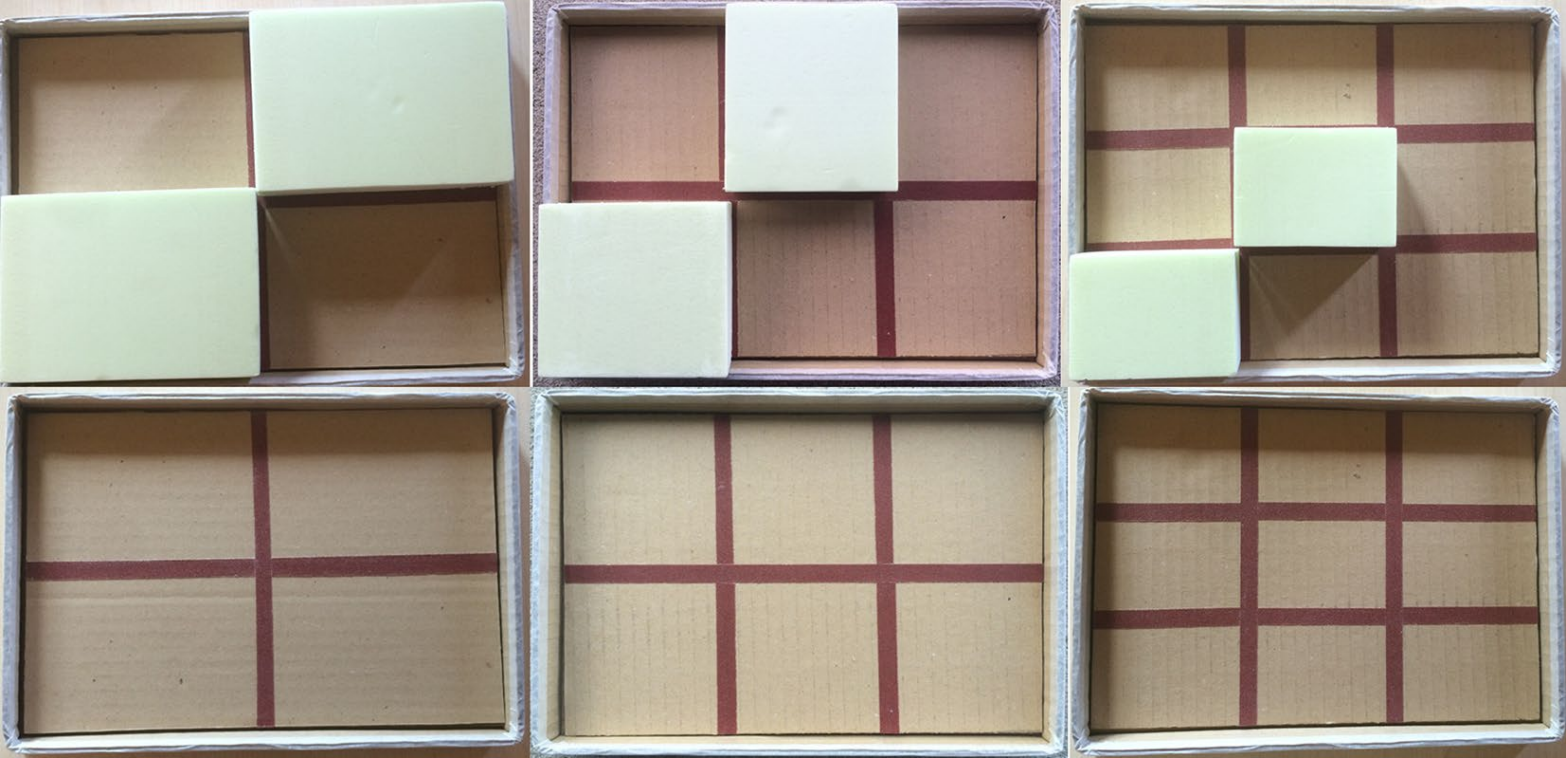
(Up) Grids, made with carton and sandpaper stripes, created to represent exactly the matrix dimensions of the virtual environment in all three Resolutions. The limits of exploration and manipulation are realized with carton borders. (Down) The same grids with also the real representations of *movable* and *fixed* objects changing in dimensions according to Resolution. From the left to the right, there are the resolutions 2 $\times$ 2, 2 $\times$ 3 and 3 $\times$ 3. The represented setup has been built by specialized rehabilitation practitioners at Fondazione Istituto Chiossone, Genoa.

Partially sighted participants were blindfolded before entering the experimental room, to prevent any visual cue. Prior to the experiments, the participants were familiarized with the task using a real setup, shown in Fig. 4 and then with the virtual environment displayed in Fig. 3 (lower part). During the familiarization, participants were asked to solve the task (task explanation is in the next paragraph) in the real setup two times. Then, the experimenter described the similarity of the real and virtual setup and let the participant interact with the *fixed* and *movable* objects. Averagely, the familiarization phase lasted about 15 min. At the beginning of each trial, the participants were told which was the position of the *fixed* and the *movable* object. They were asked to move the TAMO3 to find and explore the objects and memorize their position. The experimenter verbally informed the participant about each position using an absolute reference system associated to the matrix composition: the first part of the information about the position was relative to the row and the second to the column, e.g. down-left refers to the latest row (the closest to the participant’s body) and the most left column (left respect to the participant’s midline). An example of the instruction, for the resolution 3 $\times$ 3, is the following: “The *fixed* object is in the centre-right position, could you please reach it with the mouse? Then the *movable* object is in the top-centre position, could you please reach it with the mouse?”. Then, the experimenter requested the kind of orientation (horizontal or vertical) for the *target* parallelepiped that the participant had to build. The timing of the trial started afterwards. The *movable* object was therefore manipulated with the left hand (i.e. the NDH), using the four arrow keys of the PC keyboard. Invalid keyboard movements, such as positioning the *movable* object on/inside the *fixed* object or outside the matrix, were signalled by an audio signal from the PC, so that the participant could repeat and correct their choice. The timing was stopped when the participant informed the experimenter to have accomplished the building task.

Across the whole trial, the participant could continuously verify where the *movable* object was, by moving the TAMO3 on the graphic tablet with the DH. Therefore, the two hands could be moved independently and the perception and action sub-processes could be performed simultaneously during the timing of the trial.

A total of 1872 trials (12 participants $\times$ 3 sessions per participant $\times$ 52 trials per session) were performed. The position of the *fixed* and the *movable* object was randomized for each trial and each participant. The trials were created in order to not repeat the configuration *fixed*-*movable* intra and inter resolution. Moreover, the orientation of the *target* parallelepiped was balanced, i.e. participants were asked to create the same number of horizontal and vertical complex objects. The ideal Manhattan distance between the *fixed* and the *movable* object for each resolution was (average ± standard deviation) respectively, 0.98 ± 0.82, 1.29 ± 0.87, 1.41 ± 0.89. The average (standard deviation) values were obtained, calculating the mean (standard deviation) of all trials per all the participants.

A video of one trial is available here: https://figshare.com/s/5955272aabc7178e1b01.

At the end of the experiment, the setup usability was tested with a SUS questionnaire^64^.

### Tactile feedback

TAMO3 reproduces phalanx movements and normal fingertip deformations, respectively, with elevation and inclination cues. The mouse has a tactor (i.e., the end effector of an actuator capable of stimulating the sense of touch) that renders three tactile degrees of freedom, in each point of the virtual object, see Fig. 3 (Left). The stimulation is designed to be felt by a single finger passively resting on the tactor. The haptic feedback on the hand is composed of the kinaesthetic feedback rendered on the finger phalanxes, merged with the tactile feedback rendered on the fingertip.

In this experiment, both the *fixed* and the *movable* objects are parallelepipeds with the same height. The main tactile cue delivered by TAMO3 is then the difference in elevation between the portion of space in which there is the object (on the experimented screen: white or gray) and those in which there is not (on the experimenter screen: blue or black). The height of the objects corresponds to the tactor excursion of 15 mm, therefore when the participant was exploring the background the height was 0mm and the tactor was at the same level of the mouse shell.

### Analysis

To evaluate the performance of visually impaired people in this test, the following variables were analysed:

- the Accuracy, i.e. the percentage of *target* objects correctly built per session;
- the Execution Time, i.e. the time interval to accomplish the task for each trial of a single session;
- the Mouse Use, i.e. the relative amount of time in which the participants moved the TAMO3 on the tablet to explore the environment, with respect to the total amount of the Execution Time, expressed as a percentage;
- the Efficiency Ratio (ER), an adimensional measure to indicate the relation between the number of moves made by the participants and the minimum number of moves required to correctly build the *target* object. It is computed by:
  1 $$\begin{aligned} ER = \dfrac{1 + iMD}{1 + rMD} \end{aligned}$$
  where iMD is the ideal Manhattan Distance between the initial and the final position of the *movable* object to build the right *target* object; rMD is the real (i.e. that actually performed by the participant) Manhattan Distance between the initial and final position of the *movable* object. The Manhattan distance is the distance between two points in a grid based coordinate system on a strictly horizontal and/or vertical path (that is, along the grid lines), as opposed to the Euclidean or “as the crow flies” distance. The Manhattan distance is the sum of the horizontal and vertical components.

When the final position of the *movable* object is correct, that is when the *target* object is correct, the efficiency ratio is equal to 1. On the other side, if the *target* object is not correctly built, the efficiency ratio can be higher or lower than 1, depending on the difference between iMD and rMD. Since the positions of *movable* and *fixed* objects are randomly assigned, it could happen that no moves are necessary to form the *target* object (i.e. the ideal and real MD could be both zero in a trial), which is why the numerator and denominator must be kept higher than zero in the equation.

All the statistical analyses were performed using R software^77^. Normality of distributions was checked with the Shapiro-Wilk Normality Test, statistical comparisons were performed using general linear models (GLMs), while post-hoc comparisons were performed with Wilcoxon tests^78^ in case of categorical variables or modelled with linear regression in case of continuous variables. The p values were retained as significant after false discovery rate (FDR) correction for multiple comparisons^79^.

## Data Availability

The datasets generated and analysed during the current study are available in the Figshare repository: https://figshare.com/articles/dataset/analyses_obj_puzzle_xls/24428407.
